# Futher increase of obesity prevalence in Chinese children and adolescents - cross-sectional data of two consecutive samples from the city of Shanghai from 2003 to 2008

**DOI:** 10.1186/1687-9856-2013-S1-O49

**Published:** 2013-10-03

**Authors:** Feihong Luo, Miaoying Zhang, Fengxia Guo, Yuezhen Tu, Shuixian Shen

**Affiliations:** 1 Children's Hospital Of Fudan University, Shanghai, China; 2 The Center of Disease Control and Prevention in Xuhui District, Shanghai, China; 3 The Center of Disease Control and Prevention in Minhang District, Shanghai, China

## 

The changes of childhood obesity in Shanghai, one of the most urbanized areas in China, is believed to represent and forecast the childhood obesity prevalence in today metropolitans and future overall China. In this study, we provide estimates of the prevalence and trends of overweight and obesity among children and adolescents in Shanghai from 2003 to 2008. One urban and one suburban district were randomly selected in the study in 2003. 70,582 students in 2003 and 86,355 students in 2008 in schools of those 2 districts were examined. Data on height, weight, gender and living area were collected. Weight status was estimated by body mass index (BMI) using the International Obesity Task Force standard. The prevalence of obesity and overweight were analyzed by area, age, gender and year.

The prevalence of overweight and obesity increased significantly during the study period (2003-2008): the prevalence of overweight increased from 13.67% to 15.41% (p<0.01), prevalence of obesity increased from 3.72% to 4.58% (p<0.01). The prevalence of obesity and overweight in boys was significantly higher than that in girls (p<0.01). The prevalence of overweight and obesity in urban area was also significantly higher than that in suburb area (p<0.01). Over a 5 years period, there was a significant increase in the prevalence of obesity and overweight in children and adolescents of two districts in Shanghai. The high percentage of overweight may cause even more rapid increase in obesity in the absence of effective interventions in the near future.

